# The breast milk and childhood gastrointestinal microbiotas and disease outcomes: a longitudinal study

**DOI:** 10.1038/s41390-022-02328-w

**Published:** 2022-10-10

**Authors:** Pernilla Lif Holgerson, Anders Esberg, Christina E. West, Ingegerd Johansson

**Affiliations:** 1 Department of Odontology, Section of Pediatric Dentistry, Umeå University, Umeå, Sweden; 2 Department of Odontology, Section of Cariology, Umeå University, Umeå, Sweden; 3 Department of Clinical Sciences, Section of Pediatrics, Umeå University, Umeå, Sweden

## Abstract

**Background:**

We aimed to characterize breast milk microbiota and define associations with saliva and fecal microbiota and selected diseases in preschool children.

**Methods:**

In a longitudinal cohort study, the microbiotas from breast milk, mouth, and fecal samples were characterized by 16S rRNA gene sequencing. Questionnaires and medical records provided information on demographics, medical, and dental data.

**Results:**

The phylogeny in breast milk, saliva swabs, and feces differed at all levels (*p* < 0.0003), though all harbored species in *Streptococcus*, *Veillonella*, and *Haemophilus*. Species richness was highest in breast milk with increasing resemblance with the oral swab microbiota by increasing age. Caries-affected children at age 5 had been fed breast milk with tenfold higher abundance of caries-associated bacteria, e.g., *Streptococcus mutans*, than caries-free children (*p* < 0.002). At that age, taxa, e.g., *Neisseria sicca* were overrepresented in saliva swabs of children with otitis media (LDA score >2, *p* < 0.05). Gut symbionts, e.g., *Bacteroides*, were underrepresented in 3-month fecal samples in children later diagnosed with allergic disease (LDA score >2, *p* < 0.05).

**Conclusions:**

Distinct microbiotas for the three sources were confirmed, though resemblance between milk and oral swab microbiota increased by age. Future studies should evaluate if the observed associations with disease outcomes are causal.

**Impact:**

- Few studies have studied the association between breast milk microbiota and gastrointestinal microbiota beyond early infancy.
- The present study confirms distinct microbiota profiles in breast milk, saliva swabs, and feces in infancy and indicates increasing resemblance between breast milk and the oral microbiota by increasing age.
- The fecal microbiota at 3 months was associated with later allergic disease; the saliva microbiota by age 5 differed between children with and without otitis media at the same age; and children with caries by age 5 had been fed breast milk with a higher abundance of caries-associated bacteria.

## Introduction

The gastrointestinal microbiome develops into a complex ecosystem during the first years of life^1^ with the intestinal^1^ and oral^2^ microbial profiles in vaginally delivered infants more similar to those of their mothers than in cesarean delivered infants. In addition, the early microbiome is affected by parental and environmental transmission, exposure to antibiotics and infant feeding practices.^3–5^ Breast milk supplies energy and nutrients, crucial for the growth and development of infants, and contains bioactive factors with immunomodulatory properties^5,6^ and bacteria.^7–9^ Thus, there is support for vertical transmission of probiotic *Bifidobacterium bifidum* and *Lactobacillus gasseri* from human milk to the infant’s gut and mouth.^10–12^ Loss of beneficial *Bifidobacteria* has been associated with an increased abundance of key proinflammatory species in the gut microbiome.^13^ This finding advocates for a vital role of breastfeeding in microbiota development in the gut and likely also in the oral cavity,^14^ and there is emerging evidence that breastfeeding may influence microbial composition even beyond infancy.^15,16^ However, the few conducted studies have focused on the age span from birth to six months.^17,18^ Hence, there is a lack of knowledge regarding how the breast milk microbiota affects gastrointestinal microbiota development beyond the first six months of life. However, the child’s gastrointestinal microbial profiles develop up to around 3 years of age, a process that is affected by both external and host factors but also the programming from already established bacterial species by providing (or not) attachments sites and quorum sensing.^19^ The present study evaluated the hypothesis that breast milk microbiota associates with the gastrointestinal microbiota and some prevalent childhood diseases in a period extending beyond 6 months of age. We aimed at characterizing the microbiota in breast milk from women who had lactated for 3 months and define associations with child saliva swab and fecal microbiota at ages 3 months to 5 years as well as with selected microbiota related disease outcomes that are prevalent in childhood, namely, allergic diseases, otitis media, and dental caries.

## Materials and methods

### Subjects and sample collection

Healthy infants were consecutively recruited to a longitudinal study via the maternity ward at Umeå University Hospital, Umeå, Sweden, when screened for phenylketonuria at 2 days of age.^20^ Recruitment took place from December 2011 through April 2012. Infants whose mother had been given antibiotics in relation to delivery were not invited. For 210 infants (31% of all children born at the recruitment hospital during the inclusion period), the caregivers consented to participate with questionnaire information and breast milk, saliva swab, and fecal samples during childhood.

Breast milk was sampled by manual expression. The mothers were instructed on how to collect breast milk in a film where they were told to wash the hands and the area around the nipple with soap and water carefully and to dry the nipple thoroughly. The first expressed milk was discarded and then at least 10 mL milk was collected from both breasts. Fecal samples were collected into sterile containers using a fit-for-the-purpose spatula. All specimens were collected in the week before the next planned visit to the research clinic and immediately stored at home at -20 °C until transported frozen to the clinic for storage at −80 °C. Saliva swab samples were collected at the clinic by rubbing sterile cotton swabs against all accessible surfaces in the mouth. The swabs were swirled in buffer and stored at −80 °C.

Breast milk, feces, and saliva swab samples were collected when the infant was 3 months old. Additional saliva swab sampling took place when the child was 18 months and 3 and 5 years old, and additional feces collection took place when the child was 5 years old. Demographic, diet, dental, and medical information was obtained by questionnaires distributed to the parents at each visit.

The study was approved by the Regional Ethical Board at Umeå University, Sweden (2011-90-31M, 2016-239-32M). All parents signed informed consent to participate and that the information could be used for research.

### Medical outcomes

Information on physician-diagnosed allergic disease (eczema, food allergy, asthma, and/or allergic rhinitis) and physician-diagnosed otitis media at any time during the follow-up period was self-reported by the parent at the visit to the research clinic. Signs of dental caries (a cavity, filling or extraction due to caries) were recorded by a specialist in pediatric dentistry at the dental clinic.

### DNA extractions

Details on DNA extractions are found in Supplementary File 1. Briefly, for saliva and human milk DNA extraction, samples were thawed on ice and genomic DNA was extracted using lysozyme, mutanolysin, RNase, and Proteinase K as described previously.^21^ For feces DNA extraction, frozen stool and lysis buffer were homogenized with a mix of glass and ceramic beads, and after a series of incubations with additional lysis buffer and Proteinase K, washes, and precipitation steps, obtained DNA was dried and dissolved in TE buffer. The quality of the extracted DNA was estimated using a NanoDrop 1000 Spectrophotometer (Thermo Fisher Scientific, Uppsala, Sweden), and the quantity was estimated by a Qubit 4 Fluorometer (Invitrogen, Thermo Fisher Scientific, Waltham, MA). The same extraction protocol was applied to Milli-Q Ultrapure Water (negative control) and a mixture of known bacterial species (positive control).

### Bacteria 16S rRNA gene amplicon sequencing

16S rRNA gene amplicons (V3–V4 region) were generated (KAPA HiFi HotStart ReadyMix (2×), Wilmington, MA) by PCR (denaturing at 98 °C for 3 min; 30 cycles with denaturing at 94 °C for 20 s, annealing at 51 °C for 20 s, and extension at 72 °C for 20 s; followed by 10 min at 72 °C; and 4 °C to finish). The 341F (ACGGGAGGCAGCAG) forward and 806R (GGACTACHVGGGTWTCTAAT) reverse primers with a linker sequence, a 12 bp barcode, and the Illumina adapter were used as described by Caporaso et al.^22^ Purified pooled amplicons were adjusted to 4 nM, spiked with 5% PhiX (Illumina, the Netherlands), denatured, and diluted as described by Illumina^23^ before being loaded onto MiSeq cartridges (Illumina, San Diego, CA) and run at the Swedish Defense Research Agency research facility in Umeå, Sweden.

### 16S rRNA gene sequence analysis and taxonomic assignment

The amplicon sequences were demultiplexed using deML,^24^ paired-end reads were merged, and primers and ambiguous and chimeric sequences were removed using default settings in DADA2 within QIIME2 with the resolution of amplicon sequence variants (ASVs).^25^ Taxonomy was assigned to the ASVs using the Human Oral Microbiome Database *e*HOMD^26^ and the Greengene database.^27^ ASVs with ≥2 reads and ≥98.5% identity with a named species/unnamed phylotype were retained and those with the same taxonomic identity were aggregated.

### Data analyses

Descriptive data are presented as the means with 95% confidence limits (CIs) or proportions (%). For univariate analyses, differences were tested with nonparametric tests using SPSS version 26 (IBM Corporation, Armonk, NY). Relative abundances of taxa are expressed as the number of reads as a proportion (%) of all reads for the individual and prevalence (detection prevalence) as the number of individuals for whom a taxon was identified in a proportion of the group (%). Species shared between groups were identified in a Venn diagram^28^ where the presence of a species was defined as “yes” if it was found in ≥10% of the participants.

Multivariate analyses included nonparametric permutational multivariate analysis of variance (PERMANOVA) to evaluate differences in microbial profiles (β-diversity) using R-vegan 2.5.5 (Adonis) within QIIME2. Partial least square regression (PLS) using SIMCA P + version 15.0 (Sartorius Stedim Data Analytics AB, Malmö, Sweden) was used to evaluate taxon explanatory and predictive power in variation of allergic disease, otitis media and dental caries outcomes. The SIMCA software scales all variables to unit variance and performs K-fold cross-validation, where 1/7th of the data are systematically kept out when fitting a model on the remaining data and predicting those kept out (*Q*^2^-values). Additionally, the linear discriminant analysis effect size (LEfSe) method^29^ with logarithmic discriminant analysis (LDA) scores was used to identify taxa differing in relative abundance among allergic disease, otitis media, and dental caries status. For LEfSe analysis, Kruskal–Wallis test, an all-against-all strategy was applied with an alpha value of 0.05 and a logarithmic LDA score threshold of 2.0.

*P* values were considered statistically significant at a false discovery rate (FDR) <0.05 or presented as *q*-values when provided by the software.

## Results

### Sequencing and study group characteristics

Of the 210 infants recruited at age 2 days, 161 returned for screening at 3 months, 142 at 18 months, 146 at 3 years, and 120 at 5 years (Table 1). Dropouts were due to parental lack of time or that the child had moved from the catchment area. All infants were healthy at birth with a birth weight over 2,500 grams and a mean gestational age of 40.1 weeks. None was born before 37 weeks of pregnancy and 6 children were large for gestational age.^30^ Of the infants 152 (94%) were vaginally delivered, and 131 were fully or partially breastfed at 3 months of age. Of these, 7 children were still partially breastfed at 18 months and 3 at 3 years of age (Table 1).

**Table 1:** Characteristics of children present at each of the four visits^a^.

	3 months	18 months	3 years	5 years
	*n* = 161	*n* = 142	*n* = 146	*n* = 120
Girls, (%)	45.9	42.1	44.1	48.7
Age, months	3.1 (3.0, 3.2)	18.0 (17.8, 18.2)	35.0 (34.8, 35.3)	59.7 (59.4, 60.0)
Cesarean section, *n* (%)	9 (5.6)	9 (6.4)	8 (5.5)	7 (5.9)
Breastfed, *n* (%)	131 (82.4)	7 (5.0)	3 (2.0)	0
Body weight, kg	6.3 (6.2, 6.5)	12.3 (11.0, 13.6)	15.1 (14.7, 15.4)	20.1 (19.4, 20.8)
Body length, cm	62 (61, 62)	83 (83, 84)	96 (95, 97)	112 (111, 113)
Antibiotics^b^, *n* (%)	2 (1.2)	9 (6.5)	1 (0.7)	3 (2.5)
Probiotic drops, (%)	15.3	—	—	—
Any allergic disease^c^, (%)	—	7.0	24.0	25.0
Otitis ever 18 months to 5 years^d^	—	—	—	22.5
Caries^e^, % children	—	—	8.9	12.5

Data are presented as mean (95% CI) or proportions (%).

^a^For analyses involving sequencing data the numbers may be reduced due to that the sample sequencing did not reach a saturation criterion based on rarefaction analysis. These numbers are given in Table S1.

^b^Intake latest 3 months; none had antibiotics in the month preceding sampling.

^c^Parental-reported physician diagnosis of eczema, food allergy, asthma, and/or allergic rhinitis.

^d^Parental-reported physician diagnosis of otitis media.

^e^Dentist-determined caries.

Totally, 561 saliva swab samples were collected from the children, 119 mothers donated milk, and 77 fecal samples were collected from the infant at 3 months of age, and 50 when the child was 5 years old (Supplementary File 1 Table S1). For these 807 samples, a total of 29,117,975 sequences in 11,367 ASVs remained after quality control.

Among the 11,367 quality-controlled ASVs, 4190 matched with 98.5% identity and ≥2 sequences with an *e*HOMD sequence in 508 named or unnamed species in 17 phyla and 202 genera by *e*HOMD and the Greengenes database. Additionally, 5894 ASVs matched with 70–<98.5% identity, yielding additional reads but no genus. For 1283 ASVs, neither the *e*HOMD nor the Greengenes database found a match. For simplicity, all phylotypes are referred to as species in the text.

The number of quality-controlled reads did not reach saturation according to the rarefaction curves for some samples, including most 5-year-old fecal samples. Only samples fulfilling the saturation criterion were included in further analyses. Hence, species-level analyses included 751 samples (Supplementary File 1 Table S1). For 61 infants, both milk, swab and feces samples fulfilled the saturation criterion, and 70 children had milk and swab information from all 4 visits fulfilling this criterion. The former group was used for sensitivity analyses of results obtained when sequences from all 3-month-old infants were employed.

### Distinct microbiotas in milk, saliva swabs, and feces

**Fig. 1:**
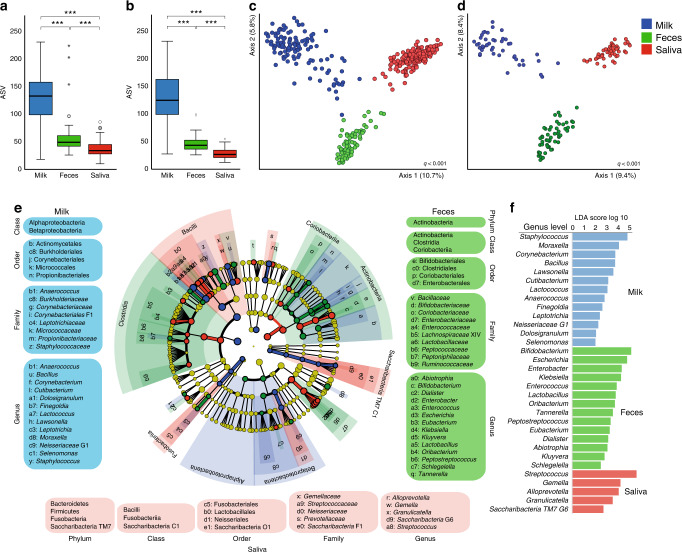
Summary of milk (blue), feces (green), and saliva swab (red) microbiota characteristics in 3-month-old infants. **a** Box-and-whisker plot of ASVs in samples from all infants with breast milk, feces or saliva swab samples; **b** for 61 infants with all three sample types. **c** Jaccard PCoA plot of microbiota profiles for samples from all 3-month-old infants and **d** for the 61 infants with all three types of samples. ***Indicates *p* values <0.001 by Kruskal–Wallis test. **e** Cladogram illustrating unique microbiota features of breast milk, feces, and saliva swab samples based on samples from all 3-month-old infants. **f** Bar plot showing LDA log^10^ scores (if >2.0) for genera that differed significantly between sample types (all 3-month-old infants).

Species richness differed significantly among milk, saliva swab, and fecal samples, with the highest number of ASVs in milk using all 3-month samples (Fig. 1a), and in the sensitivity analyses in the 61 infants with milk, saliva swab, and feces samples at the 3-month sampling (Fig. 1b). Beta-diversity by ASV detection (present/absent, Jaccard diversity index) separated the samples distinctly by source among all 3-month samples (Fig. 1c) and the 61 infants with all three sample types (Fig. 1d). Similarly, the samples were distinctly separated in PCoA plots based on diversity from taxon abundances (Bray Curtis index; *q* < 0.001), phylogeny without (unweighted UniFrac distance, *q* < 0.001) or with weighting for branch lengths (weighted UniFrac distance, *q* < 0.001) (Supplementary File 1 Fig. S1A–C). Compositional evenness also differed by sample source by the Shannon diversity index without phylogeny (*q* ≤ 1.1E−9), Faith phylogeny diversity index with phylogeny (*q* ≤ 4.3E−7) and with richness (Pielou’s evenness index) (*q* ≤ 2.6E−2) (Supplementary File 1 Fig. S2A–C). Detailed data of *e*HOMD-identified species/phylotypes in milk, oral swabs and feces are presented in Supplementary File 2 Tables S2 and S3.

### Characteristics of milk, saliva swab, and fecal microbiotas in 3-month samples

The phylogenetic characteristics of breast milk, saliva swab, and fecal microbiotas differed at all taxonomic levels, i.e., phylum, class, order, family, and genus, according to the multivariate PERMANOVA test after FDR adjustment (*p* ≤ 0.0003). LEfSe-identified features based on taxon abundances in the milk, feces, and oral swab microbiotas are presented in Fig. 1e, f and Supplementary File 2 Table S4. Thus, the breast milk microbiota was, compared with that in saliva and feces, characterized by enriched abundance in the classes Alphaproteobacteria and Betaproteobacteria, represented by the genera *Anaerococcus*, *Bacillus*, *Corynebacterium*, *Cutibacterium*, *Dolosigranulum*, *Finegoldia*, *Lactococcus*, *Lawsonella, Leptotrichia*, *Moraxella*, *Neisseriaceae* G1, *Selenomonas*, and *Staphylococcus* (LDA ≥2.0, *p* ≤ 1.04E−8) (Fig. 1e).

The saliva microbiota was, compared with that in milk and feces, characterized by enriched abundance in the phyla Bacteroidetes, Firmicutes, Fusobacteria, and Saccharibacteria (TM7), represented by the genera *Alloprevotella*, *Gemella*, *Granulicatella*, *Saccharibacteria* G6, and *Streptococcus* (LDA ≥ 2.6, *p* ≤ 5.4E−6) (Fig. 1e).

Finally, the fecal microbiota differed from that in breast milk and saliva swabs by enriched abundance in the phylum Actinobacteria represented by the genera *Abiotrophia*, *Bifidobacterium*, *Dialister*, *Enterobacter*, *Enterococcus*, *Escherichia*, *Eubacterium*, *Klebsiella*, *Kluyvera, Lactobacillus*, *Oribacterium*, *Peptostreptococcus*, *Schlegelella*, and *Tannerella* (LDA ≥ 2.5, *p* ≤ 1.01E−7) (Fig. 1e).

### Overlapping species in milk, saliva swab, and fecal microbiotas

**Fig. 2:**
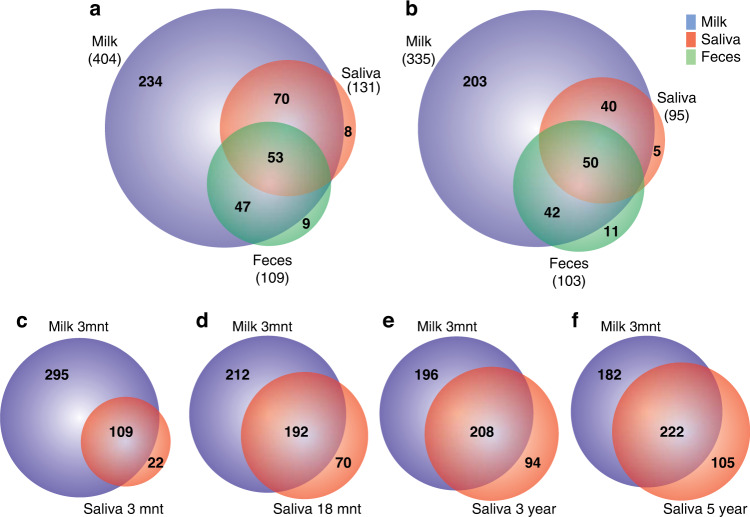
Venn diagram illustrating overlapping species among bacterial communities in milk, saliva swab, and feces. The upper panel illustrates the number of species shared among the milk (blue), saliva swab (red) and fecal (green) microbiotas in 3-month-old for **a** all samples, i.e., *n* = 116, 159, and 75, respectively, and **b** for the 61 infants with all three types of samples. The numbers in parentheses refer to the total number of species detected in the “tissue” type. The lower panel shows the number of species detected in breast milk samples from 3-month lactating mothers that were also detected in saliva swab samples collected from the same children at increasing ages, i.e., **c** 3 months (*n* = 114), **d** 18 months (*n* = 101), **e** 3 years (*n* = 92), and **f** 5 years (*n* = 82).

Species found in both the milk, saliva and fecal microbiotas at 3 months are illustrated in Venn diagrams for all available samples (Fig. 2a) and in the 61 infants with all three sample types (Fig. 2b). In both settings, 94% of saliva swab-detected taxa were present in breast milk, and 90% of *e*HOMD-detected fecal species were present in breast milk. Species present in all three types of samples were most commonly in the genera *Streptococcus* (12 species), *Veillonella* (6 species), and *Haemophilus* (6 species). Species shared among breast milk and saliva samples represented a wider array of genera with dominance of species in *Prevotella* (8 species), *Leptotrichia* (6 species), *Neisseria* (5 species), and *Porphyromonas* (4 species). Species present in both breast milk and feces were most commonly in *Corynebacterium* (7 species) and *Fusobacterium* (4 species). Complete data are presented in Supplementary File 2 Table S5A.

Of the 127 genera identified in the 3-month breast milk samples, 77 (61%) were detected in at least one saliva swab sample from 3-month-old (*n* = 114), 18-month-old (*n* = 101), 3-year-old (*n* = 92), and 5-year-old (*n* = 82) children (Supplementary File 2 Table S5A). Correspondingly, of the 404 species identified in the 3-month breast milk samples (Fig. 2a), 263 (65%) were detected in at least one saliva swab sample at the same age or later (Supplementary File 2 Table S5B). Venn diagrams illustrating the numbers of species detected in the 3-month milk samples that were also detected in saliva swab samples at each of the increasing sample ages are shown in Fig. 2c–f.

Furthermore, the temporal stability of species in breast milk and saliva swab samples at 3 months was evaluated, i.e., species potentially transmitted from breast milk and retained in the child’s mouth over time. In breast milk, 30 species were consistently found in ≥10% of children at all four swab sample ages (Supplementary File 3 Table S6). There were 6 in *Streptococcus*, 4 in *Veillonella*, 3 in *Bergeyella* and *Haemophilus*, 2 in *Gemella*, *Granulicatella*, *Neisseria*, *Porphyromonas*, and *Prevotella* and 1 in *Actinomyces*, *Alloprevotella*, *Fusobacterium*, and *Rothia*. The mean proportions of children carrying these 30 species in breast milk and saliva swabs were 64.1 and 37.2%, respectively, with corresponding 95% CIs of 56.1–72.1 and 27.2–47.3 (Supplementary File 3 Table S6).

### Associations with selected disease outcomes

Associations between taxon profiles and having a physician-diagnosed allergic disease (eczema, food allergy, asthma, and/or allergic rhinitis) and physician-diagnosed otitis media or dentist-recorded sign of dental caries were searched by multivariate PLS regression modeling (limit *R*^2^ > 0.5 and *Q*^2^ > 0.05) (Supplementary File 3 Table S7). The strongest association between ever being diagnosed with an allergic disease at 5 years was with the 3-month fecal microbiota (*R*^2^ = 0.55, *Q*^2^ = 0.26), whereas being diagnosed with otitis media by age 5 was most strongly associated with the 5-year saliva swab profile (*R*^2^ = 0.53, *Q*^2^ = 0.11). The strongest association with having caries signs at 5 years was with breast milk (*R*^2^ = 0.52, *Q*^2^ = 0.11), but an association was also seen with the saliva swab microbiota (*R*^2^ = 0.52, *Q*^2^ = 0.052).

**Fig. 3:**
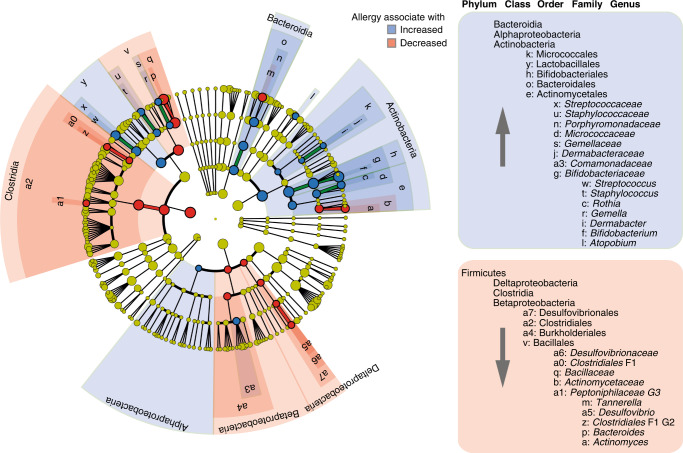
Association between the 3-month fecal microbiota and allergy development in the first 5 years of life. LEfSe analysis in the fecal microbiota at 3 months in allergic and nonallergic children. Highlighted taxa had a logarithmic LDA score >2.0 and a statistically significant difference (*p* < 0.05) by pairwise Wilcoxon test.

Specifically, the 3-month fecal microbiota of infants who developed allergic disease in the first 5 years of life (versus not) had higher abundances of the genera *Streptococcus*, *Staphylococcus*, *Rothia*, *Gemella*, *Dermabacter*, *Bifidobacterium*, and *Atopobium* and lower abundances of *Tannerella*, *Desulfovibrio*, *Clostridiales* F1 G2, *Bacteroides*, and *Actinomyces* (Fig. 3).

**Fig. 4:**
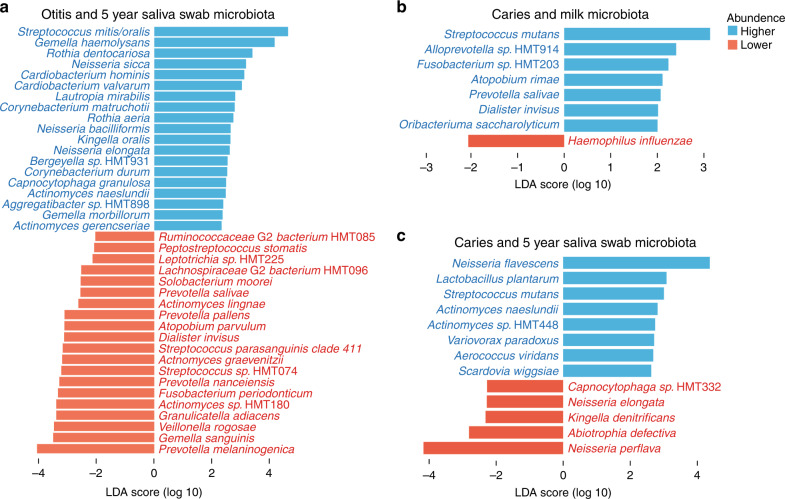
Breast milk or oral swabs microbiota at 3 months and development of otitis media or dental caries. LEfSe analysis in **a** oral swab microbiota with respect to ever having otitis media, **b** the milk microbiota and having signs of dental caries at 5 years of age, and **c** 5-year saliva swab microbiota and having signs of dental caries at 5 years of age. Bar plots indicate species association with ever having had otitis media or showing caries signs at 5 years of age. Blue denotes increased abundance, and red denotes decreased abundance in the respective microbiota. Species were selected based on logarithmic LDA score >2.0 and a statistically significant difference (*p* < 0.05) between the two groups by pairwise Wilcoxon test.

Children who had otitis by age 5 (versus not) had higher abundances of 19 species and lower abundance of 20 species in the 5-year saliva swab (Fig. 4a). The strongest positive associations were for *Streptococcus mitis*/*oralis*, *Gemella haemolysans*, *Rothia dentocariosa*, *Neisseria sicca*, *Cardiobacterium hominis*, and *Cardiobacterium valvarum*, whereas *Prevotella melaninogenica*, *Gemella sanguinis*, *Veillonella rogosae*, *Granulicatella adiascens*, and *Actinomyces sp. HMT180* appeared to be protective.

LEfSe revealed that children with caries signs at 5 years of age were fed breast milk with a higher abundance of *Streptococcus mutans*, *Alloprevotella sp. HMT914*, *Fusobacterium sp. HMT203*, *Atopobium rimae*, *Prevotella salivae*, *Dialister invisus*, and *Oribacterium saccharolyticum* but lower levels of *Haemophilus influenzae* (Fig. 4b). In univariate comparison, *S. mutans* was 10 times higher (*p* < 0.002) in breast milk in caries-affected children.

Furthermore, LEfSe indicated that children with caries signs by 5 years of age (versus not) had higher abundances of *Neisseria flavescens*, *Lactobacillus plantarum*, *Streptococcus mutans*, *Actinomyces naeslundii*, *Actinomyces sp. HMT448, Variovorax paradoxus*, *Aerococcus viridans*, and *Scardovia wiggsiae* and lower abundances of *Capnocytophaga sp. HMT332*, *Neisseria elongata*, *Kingella denitrificans*, *Abiotrophia defectiva*, and *Neisseria* in the 5-year saliva swab samples (Fig. 4c). By complementary species-specific PCR, *S. mutans* was detected in 50% (*n* = 6) of the 5-year-olds with caries and only 0.01% (*n* = 2) of caries-free children (*p* < 0.001).

## Discussion

In this prospective cohort of 3-month to 5-year-old children, we characterized the breast milk microbiota and defined associations with the oral and fecal microbiotas and selected disease outcomes. We revealed distinctly separated microbiota profiles in the 3-month breast milk, oral, and feces samples based on the beta-diversity displayed in the multivariate PCoA projection, but with increasing resemblance between the 3-months milk and the oral microbiota as the child became older. There were occasional associations between milk, oral swab, or feces microbiota and allergy, otitis media, and dental caries but causality remains unclear.

Research over the past decade using next-generation sequencing has demonstrated a diverse community of bacterial species in breast milk,^31^ although the origin is debated. The present study showed that ASV richness in breast milk from 3-month lactating mothers was markedly higher than in saliva swabs and feces from 3-month-old infants. In addition, the breast milk microbiota differed from that in saliva swabs and feces by 13 genera, including *Anaerococcus*, *Bacillus*, *Corynebacterium*, *Lactococcus*, *Leptotrichia*, *Neisseriaceae* G1, *Selenomonas*, and *Staphylococcus*. Nevertheless, all three niches contained species from the genera *Streptococcus, Veillonella*, and *Haemophilus*, previously reported to be part of a core microbiota in the milk and oral microbiomes.^17,32^

The composition of the breast milk microbiota has been reported to vary depending on lactation time and if sampled via pump or by hand.^33^ Here, the mothers collected the milk by hand, which could be why *Staphylococcus* was dominant in the milk samples; however, this finding is in line with other studies.^34,35^ *Staphylococcus* was previously reported as one of the most prevalent species in the mouth of very young infants (<1 month old),^34,35^ with specifically *S. epidermis, S. lugdunensis*, and *S. pasteuri* being prevalent in very small infants but not present from 3 months of age.^20^ Instead, the most abundant genera in the oral cavity of 3-month-old infants were *Alloprevotella*, *Gemella*, *Granulicatella*, *Saccharibacteria* G6, and *Streptococcus*. Similar to previous studies in which the oral microbiota was assessed up to 6 months in breastfed infants, we found that taxa in *Streptococcus* predominated.^18,36^ In fact, *Streptococcus* predominance in the oral microbiota has been demonstrated in newborns throughout adolescence and adulthood.^20,31,37^

The fecal microbiota differed from that in saliva and breast milk by the genera *Abiotrophia*, *Bifidobacterium*, *Dialister*, *Enterobacter*, *Enterococcus*, *Escherichia*, *Eubacterium*, *Klebsiella, Kluyvera*, *Lactobacillus*, *Oribacterium, Peptostreptococcus*, *Schlegelella*, and *Tannerella*. *Bifidobacterium*, a pioneering colonizer of the human gut, was the dominant genus at 3 months of age in this study group, which is in line with previous studies of breastfed infants.^1,38^ Breast milk contains abundant complex and structurally diverse nondigestible oligosaccharides that stimulate the growth of bifidobacteria in the infant gut.^39^ Thus, even though the microbiotas were different between breast milk and infant feces, these two bacterial communities may be intimately linked.

Although only three genera were shared among breast milk, saliva swab, and fecal samples, the overlap between milk and saliva swab samples was larger, with a dominance of species in *Prevotella*, *Leptotrichia*, *Neisseria*, and *Porphyromonas*. These results confirm and extend Oba et al.’s results in infants up to 6 months of age.^18^ Notably, the increasing resemblance between the saliva and breast milk microbiotas by age parallels the maturation of the oral microbiome in childhood. Though the source of bacteria in breast milk remains unclear, we speculate that the present finding reflects the proposed hypothesis that the milk microbiota has its origin in the mother’s oral and intestinal microbiome.^40,41^

We investigated associations between breast milk, saliva swab and feces microbiotas and selected disease outcomes, previously suggested to be associated with aberrancies in gastrointestinal tract colonization.^3,4,32,42^ There were limited disease signs for dental caries before 5 years of age, but interestingly, children who had caries at that age had been fed breast milk with higher levels of *Streptococcus mutans* and other species that may be involved in the caries process.^43,44^ This was an unexpected finding, but it is noteworthy that we previously found *S. mutans* in infants from 2 days of age.^20^ Although caries may develop in *S. mutans*-free persons,^21^ its presence is commonly found to be associated with caries development and has been suggested as a predictor for caries development.^45^ *S. mutans* in breastmilk and its association with future caries development has not been reported previously and needs to be confirmed. Here, it is interesting to notice that breast milk per se does not promote attachment of *S. mutans* to tooth tissues but affects adhesion to saliva coated hydroxyapatite in a donor dependent fashion, i.e., breast milk enhances attachment from some mothers but reduces it from others.^46^.

There is emerging interest in the role of the breast milk microbiota and the risk of developing noncommunicable diseases. In a recent study, children developing allergic diseases in the first 7 years of life had been fed breast milk (sampled at infant age 1 month) with reduced microbial richness.^47^ Though microbial richness in breast milk was not evaluated, a high explanatory power of species abundance in breast milk was found but cross-validation did not confirm prediction and the association was disregarded. However, being diagnosed with any allergic disease by 5 years of age was associated with underrepresentation of fecal *Bacteroides*, an important gut symbiont with immunomodulatory potential,^48^ confirming previous findings from us and others.^49–51^ There was also underrepresentation of *Clostridiales*, consistent with reports of reduced abundance of fecal *Clostridiales* species in allergic diseases.^52,53^ Genera that were overrepresented in the fecal microbiota of 3-month-old infants that later developed allergies also included *Bifidobacterium*. Notably*, B. infantis* was recently demonstrated to be enriched in infant feces in a rural setting compared with that in an urban setting and associated with a low risk of allergic diseases.^54^ In contrast, we and others have shown that adult-like *B. adolescentis* is associated with the development of allergic diseases.^49,55^ Collectively, bifidobacterial species have different immune-modulating capacities that can impact immune system ontogeny,^55^ but their potential role in allergy development needs to be clarified.

It is well established that breastfeeding confers protection against infections, including otitis media.^56^ We previously identified lower levels of *Moraxella catharralis* in the mouth of infants fed formula supplemented with bioactive milk fat globule membranes (MFGM) compared with standard formula,^57^ supporting that MFGM-fed infants had a lower incidence of otitis media.^58^ Here, parentally reported otitis media by age 5 was associated with the 5-year saliva profile but not with earlier saliva microbiotas or the 3-month breast milk microbiota. This finding may indicate that the association between oral bacteria, i.e., from milk or saliva, is a temporally close association rather than a long-term effect.

This study has several strengths, including the longitudinal design starting soon after birth, inclusion of healthy, term infants that were mainly vaginally delivered and breastfed at baseline, none treated with antibiotics one month before sampling, the collection of breast milk, salivary swabs and feces, and the 5-year long-term follow-up. By choosing 3 months for the milk collection the number of milk samples could be maximized, and confounding from teeth limited. Additionally, the number of drop-outs could be limited as there is a mandatory visit at the child’s health care center at 3 months. There are also possible limitations, e.g., lack of a complete set of samples from all individuals, that the sequencing results from fecal samples at 5 years of age had to be discarded because the species richness was not saturated and that we did not control for other dietary and environmental exposures that could have influenced the microbiota in the follow-up period. Several sources of errors in the 16S rDNA sequencing process, i.e., in the initial PCR amplification and the sequencing per se, may confound detection of low-frequency bacteria. Further, variations in sequence yields affects the species detection depth and thereby sample diversity and richness. The precautions taken were, beyond the filtering steps included in the DADA2 pipeline, excluding single-read ASVs, running the analyses on normalized bacterial abundances and excluding samples that did not reach saturation.

## Conclusion

In summary, the phylogenetic characteristics in breast milk, saliva swabs and feces differed at all taxonomic levels, though all three contained species from the genera *Streptococcus*, *Veillonella*, and *Haemophilus*. The resemblance of the microbiota profile in 3 months milk and the oral swab microbiota increased by increasing age of the child. Moderate associations between milk, oral swab or feces microbiota were seen with allergy, otitis media and dental caries, respectively, including that 5-year old caries-active children had been fed breast milk with 10 times more *S. mutans* than caries-free children. This may indicate that breast milk microbiota associates with later caries outcome and reflects the mothers’ *S. mutans* status. Future studies should evaluate if these associations with disease outcomes are causal.

## Supplementary information

Supplementary text

Table S4

Supplementary Table S6

## Data Availability

Sequence data are deposited at the European Nucleotide Archive (ENA) with accession number PRJEB35824.
