# Supplementary material for: The breast milk and childhood gastrointestinal microbiotas and disease outcomes: a longitudinal study
Source: Pediatr Res. 2022 Oct 10;93(3):570–8. doi: 10.1038/s41390-022-02328-w (PMC9988688; doi:10.1038/s41390-022-02328-w)

Supplementary Text, Figures and Tables for: Breast milk microbiota and child gastrointestinal microbiota and disease outcomes: A longitudinal study by Lif Holgerson et al.

Supplementary text

Saliva and human milk DNA extraction

Saliva and milk samples were thawed on ice. Genomic DNA was extracted using the GenElute™ Bacterial Genomic DNA Kit (Sigma–Aldrich, St. Louis, MO) as described previously[21]. Briefly, 150 µL of saliva swab buffer and 400 µL of milk were centrifuged for 5 minutes at 13,000 rpm; bacteria were resuspended, lysed in buffer with lysozyme and mutanolysin, and treated with RNase and Proteinase K. Released DNA was membrane bound, washed and eluted in 200 µL of elution buffer. The quality of the extracted DNA was estimated using a NanoDrop 1000 Spectrophotometer (Thermo Fisher Scientific, Uppsala, Sweden), and the quantity was estimated by a Qubit 4 Fluorometer (Invitrogen, Thermo Fisher Scientific, Waltham, MA). The same extraction protocol was applied to Milli-Q Ultrapure Water (negative control) and a mixture of known bacterial species (positive control).

Feces DNA extraction

Frozen stool (80-120 mg), lysis buffer (900 µL) and a mix of glass and ceramic beads were homogenized for 10 minutes, 50 µL of LysMix buffer was added, and samples were incubated at 37°C for 30 minutes followed by 15 minutes at 80 °C. Samples were spun at full speed for 5 minutes at 4 °C. Supernatants were transferred to clean tubes and incubated at 70 °C for 10 minutes after adding 15 µL of Proteinase K. Then, 180 µL of ammonium acetate was added, and samples were kept on ice for 5 minutes and spun at full speed for 10 minutes at 4 °C. Supernatants were transferred to DNA-free tubes and incubated on ice for 30 minutes after addition of one volume of cold isopropanol followed by spinning at full speed at 4 °C

for 20 minutes. The supernatants were discarded, the tubes were dried by tapping on paper, and precipitated DNA was washed 3 times with 500 μL of cold 70% ethanol by spinning at full speed for 5 minutes at 4 °C. The obtained DNA was dried and dissolved in 200 μL of TE buffer. The quality and quantity of extracted DNA were estimated as described above. The same person performed all milk, saliva and fecal DNA extractions.

Supplementary Table S1. Summary of attending participants, samples obtained and sequenced, and samples fulfilling saturation as displayed in rarefaction curves. The numbers refer to *e*HOMD recognized species/ phylotypes at ≥98.5 identity and with ≥2 reads.

	3 months	18 months	3 years	5 years
**Attended visit, n**	161	142	146	120
**Sequenced milk samples**				
Total number of samples[a], n	119	—	—	—
Samples with saturation, n	116			
N reads	4,044,848	—	—	—
Saturation at read number	4,400	—	—	—
Minimum reads	4,444	—	—	—
Mean reads	33,990	—	—	—
**Sequenced oral swab samples**				
Total number of samples[b], n	159	142	141	119
Samples with saturation, n	159	141	141	119
N reads	6,873,423	3,578,974	4,254,605	8,392,798
Saturation at read number	1,100	2,200	2,200	4,400
Minimum reads	12,925	8,166	13,098	21,071
Mean reads	43,229	25,383	30,175	70,528
**Sequenced feces samples**				
Total number of samples[c], n	77	—	—	50
Samples with saturation, n	75	—	—	—
N reads	1,836,075	—	—	—
Saturation at read number	1,100	—	—	—
Minimum reads	1,849	—	—	—
Mean reads	24,481	—	—	—

a) 52 care-givers (32%) did not return a milk sample.

b) 2, 5, and 1 child(ren) did not cooperate at the 3 months, 3 years and 5 years saliva-swabbing. sampling, respectively.

c) 84 care-givers (48%) did not return a feces sample when the child was 3 months old and 70 care-givers (58%) did not did not return a feces sample when the child was 5 years old.

Supplementary Figure S1. PCoA plots for (**A**) Bray Curtis, and (**B**) unweighted and (**C**) weighted unifrac distances for milk (blue), oral swab (red) and feces (green) samples using all available samples at 3 months. FDR adjusted p-values (q) are shown for all models.

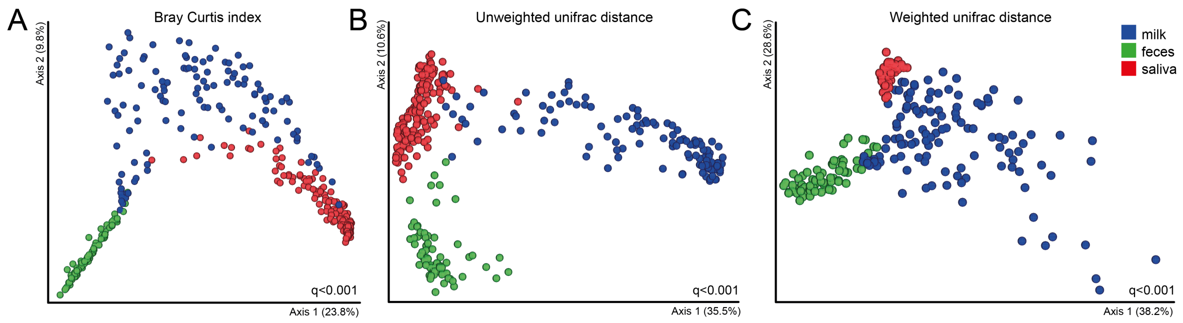

Supplementary Figure S2. Box-and-whisker plots showing median values with quartile limits of **(A)** Shannon diversity index, (**B**) Faith phylogeny diversity index and (**C**) Pielou´s Evenness index for milk (blue), feces (green) and saliva (red) samples using all available samples at 3 months.

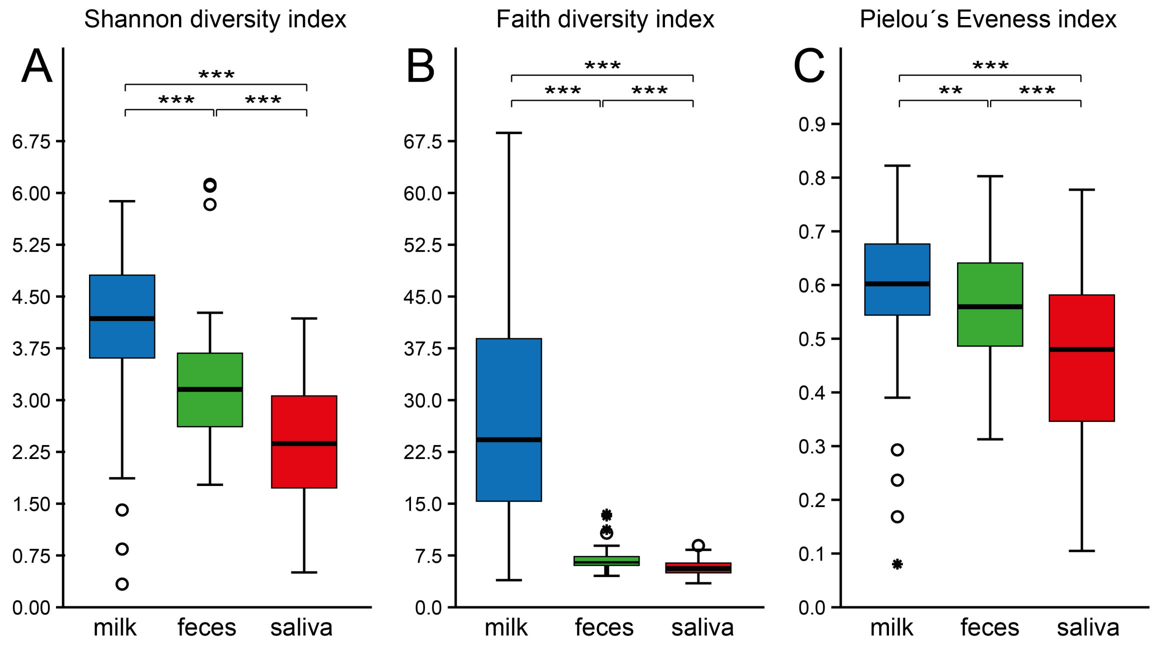

Supplement: Supplementary file 1 — Supplementary text [file 41390_2022_2328_MOESM1_ESM.pdf]
