# Supplementary material for: The breast milk and childhood gastrointestinal microbiotas and disease outcomes: a longitudinal study
Source: Pediatr Res. 2022 Oct 10;93(3):570–8. doi: 10.1038/s41390-022-02328-w (PMC9988688; doi:10.1038/s41390-022-02328-w)
Supplement: Supplementary file 3 — Supplementary Table S6 [file 41390_2022_2328_MOESM3_ESM.pdf]

Supplementary Figures and Tables for: The breast milk and childhood gastrointestinal microbiotas and disease outcomes: A longitudinal study by Lif Holgerson et al.

Supplementary Table S6. Species in breast milk at 3 months of age and also present in saliva swab samples in at least 10% of the infants at all four sampling occasions. The numbers represent the mean proportions (%) of all breast milk samples and merged saliva swab samples where the species was found.

Species	Breast milk	Saliva swab
Actinomyces sp. HMT 180	85,7	14,3
Alloprevotella sp. HMT 473	56,8	45,7
Bergeyella sp. HMT 206	75	12,9
Bergeyella sp. HMT 322	27,3	15,7
Bergeyella sp. HMT 931	41,5	21,4
Fusobacterium periodonticum	53,8	18,6
Gemella haemolysans	92,4	97,1
Gemella sanguinis	58,8	17,1
Granulicatella adiacens	78,9	27,1
Granulicatella elegans	68,3	55,7
Haemophilus parainfluenzae	80,5	58,6
Haemophilus sp. HMT 036	68,3	57,1
Haemophilus sp. HMT 908	30,8	10
Neisseria cinerea	61,5	20
Neisseria perflava	41,7	14,3
Porphyromonas pasteri	60	28,6
Porphyromonas sp. HMT 930	58,3	50
Prevotella melaninogenica	66,7	24,3
Prevotella nanceiensis	33,3	17,1
Rothia mucilaginosa	92,1	88,6
Streptococcus australis	44,7	31,4
Streptococcus mitis_oralis	100	100
Streptococcus parasanguinis clade 411	84,6	15,7
Streptococcus salivarius	94,5	71,4
Streptococcus sp. HMT 066	50	17,1
Streptococcus sp. HMT 074	26,9	40
Veillonella atypica	71,4	12,9
Veillonella dispar	94,5	55,7
Veillonella parvula	50	10
Veillonella sp. HMT 780	75,8	67,1

Supplementary Table S7. Summary of PLS derived explanatory (R2) and predicitive (Q2) coefficients.

	**3 months**		**18 months**		**3 years**		**5 years**	
	R^2	Q^2	R^2	Q^2	R^2	Q^2	R^2	Q^2
**Allergy**								
Milk abundance	0,64	-0,13	nt		nt		nt	
Milk dikotom	0,61	0,03	nt		nt		nt	
Swab abundance	0,64	-0,13	0,63	-0,03	0,46	0,07	0,48	0,05
Swab dikotom	0,61	0,03	0,59	-0,07	0,59	0,08	0,61	0,09
Feces abundance	0,55	0,26	nt		nt		0,84	-0,18
**Otitis**								
Milk abundance	0,53	-0,13	nt		nt		nt	
Milk dikotom	0,68	-0,11	nt		nt		nt	
Swab abundance	0,35	0,01	0,62	-0,21	0,53	-0,12	0,53	0,11
Swab dikotom	0,33	-0,20	0,61	-0,21	0,60	-0,11	0,65	0,12
Feces abundance	nt		nt		nt		nt	
**Caries**								
Milk abundance	0,52	0,11	nt		nt		nt	
Milk dikotom	0,58	-0,07	nt		nt		nt	
Swab abundance	0,36	-0,14	0,44	-0,09	0,49	-0,07	0,52	0,05
Swab dikotom	0,40	-0,09	0,54	-0,11	0,67	-0,11	0,65	0,08
Feces abundance	nt		nt		nt		nt	
